# BIM Mediates EGFR Tyrosine Kinase Inhibitor-Induced Apoptosis in Lung Cancers with Oncogenic EGFR Mutations 

**DOI:** 10.1371/journal.pmed.0040315

**Published:** 2007-10-30

**Authors:** Daniel B Costa, Balázs Halmos, Amit Kumar, Susan T Schumer, Mark S Huberman, Titus J Boggon, Daniel G Tenen, Susumu Kobayashi

**Affiliations:** 1 Division of Hematology/Oncology, Beth Israel Deaconess Medical Center, Harvard Medical School, Boston, Massachusetts, United States of America; 2 University Hospitals of Cleveland and Case Western Reserve University, Cleveland, Ohio, United States of America; 3 Department of Pharmacology, Yale University School of Medicine, New Haven, Connecticut, United States of America; University of California Los Angeles, United States of America

## Abstract

**Background:**

Epidermal growth factor receptor (EGFR) mutations are present in the majority of patients with non-small cell lung cancer (NSCLC) responsive to the EGFR tyrosine kinase inhibitors (TKIs) gefitinib or erlotinib. These EGFR-dependent tumors eventually become TKI resistant, and the common secondary T790M mutation accounts for half the tumors with acquired resistance to gefitinib. However, the key proapoptotic proteins involved in TKI-induced cell death and other secondary mutations involved in resistance remain unclear. The objective of this study was to identify the mechanism of EGFR TKI-induced apoptosis and secondary resistant mutations that affect this process.

**Methods and Findings:**

To study TKI-induced cell death and mechanisms of resistance, we used lung cancer cell lines (with or without *EGFR* mutations), Ba/F3 cells stably transfected with *EGFR* mutation constructs, and tumor samples from a gefitinib-resistant patient. Here we show that up-regulation of the BH3-only polypeptide BIM (also known as BCL2-like 11) correlated with gefitinib-induced apoptosis in gefitinib-sensitive *EGFR*-mutant lung cancer cells. The T790M mutation blocked gefitinib-induced up-regulation of BIM and apoptosis. This blockade was overcome by the irreversible TKI CL-387,785. Knockdown of BIM by small interfering RNA was able to attenuate apoptosis induced by EGFR TKIs. Furthermore, from a gefitinib-resistant patient carrying the activating L858R mutation, we identified a novel secondary resistant mutation, L747S in cis to the activating mutation, which attenuated the up-regulation of BIM and reduced apoptosis.

**Conclusions:**

Our results provide evidence that BIM is involved in TKI-induced apoptosis in sensitive *EGFR*-mutant cells and that both attenuation of the up-regulation of BIM and resistance to gefitinib-induced apoptosis are seen in models that contain the common EGFR T790M and the novel L747S secondary resistance mutations. These findings also suggest that induction of BIM may have a role in the treatment of TKI-resistant tumors.

## Abstract 2

Susumu Kobayashi and colleagues provide evidence that the polypeptide BIM is involved in tyrosine kinase inhibitor (TKI)-induced apoptosis in sensitive*EGFR*-mutant cells and suggest that induction of BIM may have a role in the treatment of TKI-resistant tumors.

## Editors' Summary

**Background.:**

Most cases of lung cancer—the leading cause of cancer deaths worldwide—are “non-small cell lung cancer” (NSCLC). Many patients with NSCLC die within a year of their diagnosis, but recently, “targeted” therapies have increased the life expectancy of some of them. Like all cancers, NSCLC occurs when cells begin to divide uncontrollably because of changes (mutations) in their genes. Targeted therapies specifically attack these changes and, unlike standard chemotherapy drugs, kill cancer cells without damaging normal cells. The targeted drugs used to treat NSCLC are gefitinib and erlotinib, two epidermal growth factor receptor (EGFR) tyrosine kinase inhibitors (TKIs). In normal cells, messenger proteins bind to EGFR and activate its tyrosine kinase, an enzyme that sticks phosphate groups on tyrosine (an amino acid) in other proteins. These “phosphorylated” proteins then tell the cell to divide. In some NSCLCs, EGFR drives uncontrolled cell division because its tyrosine kinase is mutated and the cancer becomes dependent on or “addicted” to EGFR signaling for its survival. TKI treatment can dramatically shrink this subset of NSCLCs, most of which lack a specific part of *EGFR* (the gene that encodes EGFR) or have the amino acid leucine instead of arginine at position 858 (an L858R mutation) of EGFR.

**Why Was This Study Done?:**

TKI-sensitive NSCLCs eventually become resistant to TKIs because they acquire additional (secondary) mutations. In half of these TKI-resistant tumors, the additional mutation is replacement of threonine by methionine at position 790 (T790M) in EGFR. However, the mutations responsible for the remaining cases of TKI resistance are not known. In addition, little is known about how TKIs induce cell death other than that they induce a type of cell death called apoptosis. A better understanding of how TKIs kill tumor cells and how secondary mutations block their effects could reveal ways to enhance their action and improve the outcome for patients with NSCLC. In this study, the researchers have studied the mechanism of TKI-induced cell death and of resistance to TKIs.

**What Did the Researchers Do and Find?:**

The researchers first measured the ability of gefitinib to cause apoptosis (genetically programmed cell death) in NSCLC cell lines (tumor cells adapted to grow indefinitely in dishes) that had the *EGFR* deletion, the L858R mutation, or normal EGFR. Gefitinib caused apoptosis only in cell lines with altered EGFR. Then they asked whether a proapoptotic protein called BIM (a member of the BCL2 family of pro- and antiapoptotic proteins) is involved in TKI-induced cell death—BIM is known to be involved in this process in leukemia (blood cancer) cells. Gefitinib treatment increased the expression of BIM in TKI-sensitive NSCLC cell lines and reduced the phosphorylation of BIM (which makes BIM more active). By contrast, blocking BIM expression using a technique called RNA interference reduced TKI-induced apoptosis in TKI-sensitive NSCLC cells. Furthermore, introduction of the T790M resistance mutation into these cells blocked gefitinib-induced up-regulation of BIM and apoptosis. Finally, the researchers identified a new TKI resistance mutation (L747S, substitution of serine for leucine at position 747) in a patient whose TKI-sensitive NSCLC had become resistant to gefitinib, and showed that this resistance mutation also reduced TKI-induced apoptosis in cells growing in dishes by interfering with BIM up-regulation.

**What Do These Findings Mean?:**

These findings (and those reported by Gong et al. and Cragg et al.) show that BIM is required for TKI-induced apoptosis in *EGFR* mutant NSCLC cells. They also show that mutations that make TKI-sensitive cells resistant to these drugs reduce TKI-induced apoptosis by preventing the upregulation of BIM. These results were obtained by examining the behavior of established cell lines growing in dishes and need to be confirmed in cells freshly isolated from tumors and in tumors themselves. However, they suggest that the efficacy of TKIs could be increased by finding ways to increase BIM expression or to activate other proteins involved in apoptosis Such approaches might be particularly beneficial for patients with NSCLC whose initially TKI-sensitive tumors have acquired mutations that make them resistant to TKIs.

**Additional Information.:**

Please access these Web sites via the online version of this summary at http://dx.doi.org/10.1371/journal.pmed.0040315.

- Ingo Mellinghoff discusses this paper and two related ones in a perspective article
- US National Cancer Institute information for patients and professionals on lung cancer (in English and Spanish)
- Information for patients from Cancer Research UK on lung cancer, including information on treatment with TKIs
- CancerQuest information on all aspects of cancer from Emory University (in several languages)
- Wikipedia pages on apoptosis, epidermal growth factor receptor, and BCL2 proteins (note that Wikipedia is a free online encyclopedia that anyone can edit; available in several languages)
- Information for patients from Cancerbackup on erlotinib and gefitinib

## Introduction

Sequencing of the *epidermal growth factor receptor* (*EGFR*) gene in a large number of tumor samples has identified somatic activating mutations in the tyrosine-kinase pocket of EGFR [1,2]. These mutations were first described in non-small cell lung cancer (NSCLC) patients treated with specific EGFR tyrosine kinase inhibitors (TKIs)—gefitinib and erlotinib—who had radiographic and clinical responses to such agents [3–5]. Two recent transgenic mouse models, in which the overexpression of *EGFR* mutations was targeted in type II pneumocytes, demonstrated that these mutations led to the development of adenocarcinomas and that the tumors responded both to suppression of the EGFR driving signal and EGFR TKIs [6,7]. As data accumulate, it seems clear that *EGFR*-mutant “oncogene-addicted” cancers represent a distinct form of NSCLC that can be targeted through novel approaches [8]. The tumor cells are dependent on, or addicted to, the *EGFR* mutated oncogene for both maintenance of the malignant phenotype and cell survival. At the time of this writing, phase II trials in which patients with advanced NSCLC are included on the basis of presence of the two most common *EGFR* mutations (either exon 19 deletions or the exon 21 arginine-for-leucine substitution at amino acid 858, or L858R) and are given gefitinib as first-line treatment show radiographic response rates that exceed 75% [9–11]. Mature results of such trials will likely confirm the improved time to progression and survival seen in retrospective studies of patients treated with TKIs in which *EGFR* mutations had been identified [12–15].

Despite the unprecedented responses seen in these specific *EGFR*-mutant tumors, most eventually become resistant to the TKIs and disease progression is noted. Our group and others have identified a second mutation in the EGFR kinase domain (the exon 20 methionine to threonine substitution at position 790, or T790M) in repeat tissue samples from patients who initially responded to TKIs but later progressed [16,17]. The two largest cohorts of patients with TKI-resistant NSCLCs, in which a second biopsy was obtained after progression, identified the T790M mutation in around 50% of the samples and one D761 secondary mutation [18,19]. Recently, in four out of 18 (22%) TKI-resistant *EGFR*-mutant tumors, amplification of another oncogene, *MET*, was identified [20]. Other secondary mutations and alternative mechanisms of resistance have not been completely clarified.

One of the major effects of TKIs in sensitive *EGFR*-mutant cell lines is their induction of apoptosis. The exquisite sensitivity of these NSCLCs to gefitinib and erlotinib [3–5] has been supported by the concept of “oncogene addiction” [6,7,21]. A recent report suggested that a common signaling cascade may be involved during apoptosis in cells that depend on oncogenic *SRC*, *BCR-ABL*, and mutant *EGFR* [22]. Interestingly, the BH3-only proapoptotic proteins BIM (also referred to as BCL2-like 11, or BCL2L11), and to a lesser extent BAD (BCL2 antagonist of cell death), mediate imatinib-induced apoptosis of *BCR-ABL* leukemic cells [23]. The key downstream mediators of TKI-induced cell death in *EGFR*-mutant tumors remain unknown. We hypothesized that the BH3-only members might be involved in the apoptotic signal following EGFR disruption by TKIs.

In this study we studied BIM's role in TKI-induced apoptosis in *EGFR*-mutant lung cancers. In addition, we investigated the effect of the resistant mutation T790M and a novel secondary mutation, L747S, on the regulation of BIM and apoptosis.

## Methods

### Patient Characteristics and Clinical Course after TKI Treatment

Two *EGFR* mutation-positive patients with gefitinib-resistant NSCLCs and secondary *EGFR* mutations were identified from our Thoracic Oncology Clinic database. Their clinical and molecular characteristics, as well as their response to TKI treatment, are detailed in Table S1. Both patients are part of an Institutional Review Board-approved protocol, and written informed consent was obtained for the analysis of their tumors.

### Reagents

Gefitinib and erlotinib were purchased from a commercial supplier. CL-387,785 was purchased from Calbiochem (Darmstadt, Germany). Stock solutions for gefitinib, erlotinib, and CL-387,785 were prepared as previously described [16].

### Sequencing of the *EGFR* Gene

Both genomic DNA and total RNA were extracted from the tumor cells of a transbronchial biopsy and of pleural fluid in patients 1 and 2 (Table S1), respectively. Genomic DNA was used as a template for sequencing exons 18–21 as previously published [3]. cDNA was transcribed from 1 μg of total RNA with Superscript II Reverse Transcriptase (Invitrogen, Carlsbad, CA). The cDNA was used as a template for subsequent PCR amplifications of *EGFR*. The kinase domain of the *EGFR* coding region was amplified by the use of two sets of oligonucleotides and sequenced: (1) sense primer (5′-GCA CAG GAC GGG GAC CAG ACA ACT-3′) and antisense primer (5′-GGA CAT AGT CCA GGA GGC AG-3′); (2) sense primer (5′-GCA CAG GAC GGG GAC CAG ACA ACT-3′) and antisense primer (5′-ATG GGT GGC TGA GGG AGG CGT TCT-3′). The PCR products containing exons 19–21 amplified by the use of the latter set of primers were subcloned into the pGEM-T Easy cloning vector (Invitrogen) and sequenced [16].

### Cell Culture

Ba/F3 cell lines were maintained in RPMI supplemented with 10% FBS and 5% WEHI conditioned medium as the source of IL3. The human lung cancer-derived cells lines A549, NCI-H460 (H460), NCI-H1975 (H1975), NCI-H3255 (H3255), PC-9, and HCC827 were maintained in RPMI supplemented with 10% FBS.

### EGFR Mutant Constructs and Transfections

The L747S mutation was introduced into human EGFR wild-type (WT) or L858R constructs in the context of the pcDNA3.1 expression vector (Invitrogen) [16] using the QuikChange XL Site-Directed Mutagenesis Kit (Stratagene, La Jolla, CA). The oligonucleotides sequences were as follows: sense primer, 5′-CGT CGC TAT CAA GGA ATC AAG AGA AGC AAC ATC TC-3′; antisense primer, 5′-GAG ATG TTG CTT CTC TTG ATT CCT TGA TAG CGA CG-3′. The resulting constructs were confirmed by sequencing. For transient transfection experiments, COS-7 cells were plated at a concentration of 5 × 10^4^ cells per well in six-well plates. The following day, these cells were transfected with 1 μg of the expression constructs using Fugene 6 (Roche) and incubated for 12 h when the medium was changed to serum-free. After 12 h of serum starvation, cells were stimulated with 100 ng/ml EGF (Sigma). TKIs were added to the culture medium 3 h prior to the addition of EGF. Cells were exposed to EGF for 15 min. Stable Ba/F3 cell and HCC827 cell lines carrying WT or other mutant EGFR were generated and maintained as previously described [24,25].

### Western Blotting and Antibodies

Whole-cell lysates were prepared as previously described [16,26]. The human lung cancer cells lines were treated in RPMI supplemented with 10% FBS in the presence of EGFR inhibitors as indicated. Ba/F3 cells were washed three times with RPMI only and stimulated by EGF as previously described [24]. Gefitinib, erlotinib, or CL-387,785 at increasing concentrations were added to the medium as indicated in the figure legends.

EGFR, caspase-3, BCL-x_L_, Bcl2 (mouse specific), and total STAT5 antibodies were purchased from Santa Cruz Biotechnology (Santa Cruz, CA). Total extracellular signal-regulated protein kinase (ERK) antibody was purchased from BD Transduction Laboratories (Lexington, KY). Phospho-EGFR (pTyr1068), phospho-STAT5 (pTyr694), phospho-AKT (pS473), phospho-ERK1/2 (pT202/pY204), BCL2 (human specific), BIM, BAD, phospho-BAD (pS112), poly (ADP-ribose) polymerase (PARP), cleaved-PARP, and AKT antibodies were purchased from Cell Signaling Technology (Beverly, MA). BIM antibody was also purchased from Stressgen (Victoria, Canada). Actin antibody was purchased from Sigma (St. Louis, MO).

### Cell Proliferation Assay

Cell counts were performed at daily intervals using Trypan blue dye exclusion. Growth inhibition was assessed by CellTiter 96 AQueous One solution proliferation kit (Promega, Madison, WI) [24]. Briefly, Ba/F3 stable cells were washed three times with RPMI 1640 only and resuspended in RPMI 1640 supplemented with 10% FBS and 20 ng/ml EGF (Sigma, St. Louis, MO). Then, cells were transferred to triplicate wells at 10,000 cells/well in 96-well flat-bottom plates with various concentrations of inhibitors and the cells were incubated for 48 h.

### Flow Cytometric Analysis of Cell-surface Exposure of Phosphatidylserine and Mitochondrial Membrane Potential

For flow cytometric analysis, cells were plated at 1 × 10^5^/well in six-well plates and treated with DMSO or EGFR inhibitors. Cell-surface exposure of phosphatidylserine was assessed using an Annexin-V-FLUOS staining kit (Roche, Basel, Switzerland) as previously described [24]. For assessment of mitochondrial membrane potential, Ba/F3 cells were incubated with 40 nM DiOC6(3) (Molecular Probes, Eugene, OR) in PBS for 15 min at room temperature as previously described [27].

### RNA Interference

*BIM*-specific and negative control small interfering RNAs (siRNAs) were purchased from Cell Signaling Technology and Dharmacon Research (Lafayette, CO), respectively. Cells were transfected with TransIT-TKO transfection reagent (Mirus, Madison, WI) according to the manufacturer's protocol in the presence of siRNAs. After 24 h of transfection, cells were washed with RPMI twice and incubated with RPMI containing 10% FBS in the presence of DMSO (control), gefitinib, or CL387,785 for 48 h.

### Statistical Analysis

The paired Student t-test was used to determine statistical significance. A *p*-value less than 0.01 was considered significant.

## Results

### Apoptosis in NSCLCs with and without *EGFR* Mutations

We selected a set of NSCLC cell lines to identify the differential sensitivity of wild-type (WT) *EGFR* and mutant tumors to the apoptotic effects of gefitinib. A549 and H460 have WT EGFR and are highly resistant to gefitinib (reported gefitinib sensitivity for proliferation [IC_50_] are 9.6 and 12.9 μM, respectively) [28], whereas H1650 and HCC827 have a deletion in exon 19 (delE746-A750) with different gefitinib sensitivities (IC_50_: 1 and 0.005 μM, respectively) [28]. H3255 carries the L858R *EGFR* exon 21 point mutation and has an IC_50_ of 0.015 μM to gefitinib [28]. In addition, we also tested PC-9 cells, which have the delE746-A750 *EGFR* mutation and are sensitive to reversible EGFR tyrosine kinase inhibitors [22].

**Figure 1:**
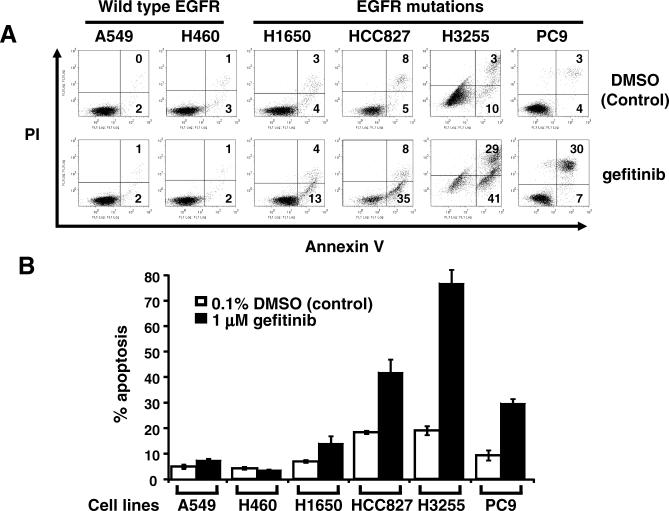
Up-Regulation of BIM Correlates with Gefitinib-Induced Apoptosis in NSCLC Cell Lines Effect of gefitinib on NSCLC cells. A549, H460, H1650, HCC827, H3255, or PC-9 cells were grown in the presence of 0.1% DMSO (control) or 1 μM gefitinib for 48 h. Apoptosis was assessed using propidium iodide and Annexin-V staining. (A) Representative flow cytometry data. The numbers represent percentage of cells in the appropriate quadrant. Left lower quadrant, viable cells; right lower quadrant, early apoptotic cells; right upper quadrant, late apoptotic cells. (B) Quantification of apoptosis. The y-axis plots the sum of early and late apoptotic cells as mean ± standard error of the mean (*n* ≥ 3).

After 48 hours of 1 μM gefitinib treatment, all the *EGFR* mutant cell lines showed an increase in the percentage of apoptotic cells when compared to untreated cells (Figure 1). However, the HCC827, H3255, and PC-9 cells had a greater increase in the amount of apoptotic cells after treatment than did the H1650 cells. The non-*EGFR* mutant A549 and H460 cells displayed almost no changes in the number of apoptotic cells after gefitinib treatment (Figure 1).

### Up-Regulation of BIM in *EGFR*-Mutant NSCLCs Sensitive to Gefitinib

Knowledge of the differential degrees of apoptotic induction by gefitinib in these cell lines prompted us to test our hypothesis that the BH3-only BIM is involved in the cell death execution process mediated by TKIs. The *BIM* gene encodes three major isoforms: BIM short (BIM_s_), BIM long (BIM_L_), and BIM extra long (BIM_EL_). All isoforms contain a BH3 domain that can bind to and inactivate members of the antiapoptotic BCL2 family of proteins [29].

**Figure 2:**
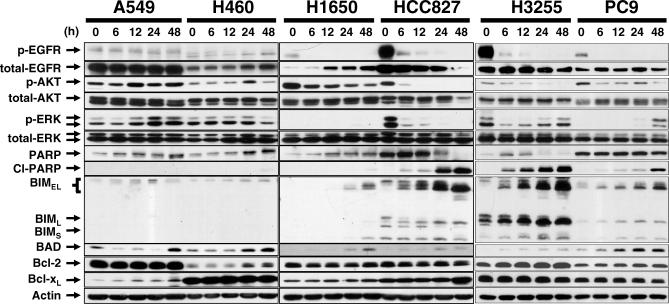
Gefitinib Induces Expression and Dephosphorylation of BIM in Gefitinib-Sensitive *EGFR*-Mutant Cells, but Not in Cells with Wild-Type *EGFR* Cells were treated with 1 μM gefitinib for the times (in hours) indicated, and lysates were collected and proteins analyzed by immunoblotting. The three isoforms of BIM, BIM_EL_, BIM_L_, and BIM_S_ are shown. Hypophosphorylated BIM migrates faster than the other species [23].

As shown in Figure 2, gefitinib treatment induced rapid and sustained increase in the levels of BIM_EL_ and BIM_L_ in HCC827, H3255 and PC-9. Two major pathways regulate BIM expression and/or function: the PI3K-AKT-FOXO and the ERK1/2 mitogen-activated protein kinase (MAPK) pathways [29,30]. Consistent with these reports, we detected that the relative electrophoretic migration BIM_EL_ and BIM_L_ was faster when HCC827, H3255, and PC-9 cells were treated with gefitinib, which suggests that BIM_EL_ and BIM_L_ were hypophosphorylated due to loss of AKT and/or ERK activity (Figure 2), or other EGFR downstream targets. In contrast, there was no sign of hypophosphorylation or marked up-regulation of BIM in A549 and H460 cells, possibly because of sustained phosphorylation of AKT/ERK (Figure 2). In H1650 cells, there was a slight increase in the level of BIM_EL_ (Figure 2), consistent with the degree of apoptosis seen in these cells (Figure 1). Changes in the proapoptotic BAD and the antiapoptotic proteins BCL2 and BCL-x_L_ did not correlate with cell death upon gefitinib exposure (Figure 2).

These findings suggest that BIM may be a key marker or effector of gefitinib-induced apoptosis in *EGFR*-mutant lung cells.

### The T790M Secondary Resistant Mutation Abrogates the Up-Regulation of BIM by Reversible TKIs

If an increase in BIM expression is important for gefitinib-induced apoptosis, it should be suppressed upon treatment of cell lines expressing the resistant T790M *EGFR* mutation [16,17] with gefitinib. We tested this hypothesis in gefitinib-sensitive HCC827 stable cell lines expressing activating deletion mutant L747-S752 (HCC/Del) *EGFR* or in the gefitinib-resistant lines HCC827-delL747-S752-T790M (HCC/Del-TM), harboring both the delL747-S752 and T790M mutations [25], as well as the H1975 cell line harboring L858R-T790M double mutations [24,25].

**Figure 3:**
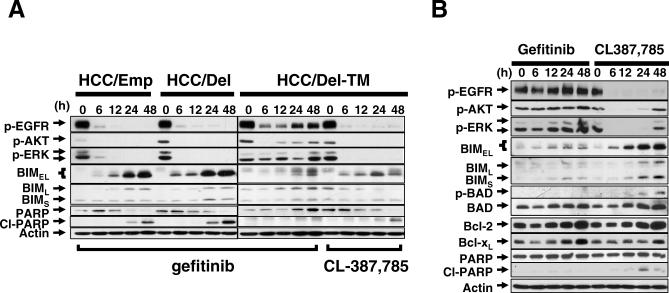
Inhibition of BIM Up-Regulation by the Resistant T790M Mutation The increase in BIM expression and apoptosis are inhibited by the secondary resistant mutation, T790M. (A) Modulation of signaling following gefitinib treatment in HCC827 cells expressing EGFR mutants. Time course of gefitinib treatment in HCC827 cells expressing pcDNA3.1 empty vector (HCC/Emp), EGFR-DelL747-S752 (HCC/Del), or EGFR-DelL747-S752+T790M (HCC/Del-TM). Cells were treated with 3 μM gefitinib or CL-387,785 for indicated times, lysates collected, and proteins analyzed by immunoblotting. (B) Modulation of signaling following either gefitinib or CL387,785 treatment in H1975 cells. The cells were treated with 1 μM gefitinib or CL-387,785 for the indicated times and extracts were analyzed by Western blotting.

Gefitinib induced rapid inactivation of EGFR, AKT, and ERK, and a dramatic increase of BIM in both HCC827 with an empty vector (HCC827/Emp) and HCC/Del cells (Figure 3A). In contrast, the HCC/Del-T790M and H1975 cells had minimal up-regulation of BIM, and the EGFR signaling cascade was less inhibited by gefitinib (Figure 3). Previously, we showed that the irreversible EGFR inhibitor, CL-387,785, can overcome gefitinib resistance and lead to apoptosis in HCC/Del-T790M [25] and H1975 cells [24]. After exposure to CL-387,785 a decrease in phosphorylations of EGFR, AKT, and ERK were observed and accompanied by marked up-regulation of BIM, which correlated with the presence of the cleaved form of PARP (Figure 3).

These results confirm that up-regulation of BIM correlates with effective TKI-induced apoptosis and that T790M suppresses this process in gefitinib-treated cells.

### Knockdown of *BIM* Attenuates TKI-Induced Apoptosis

**Figure 4:**
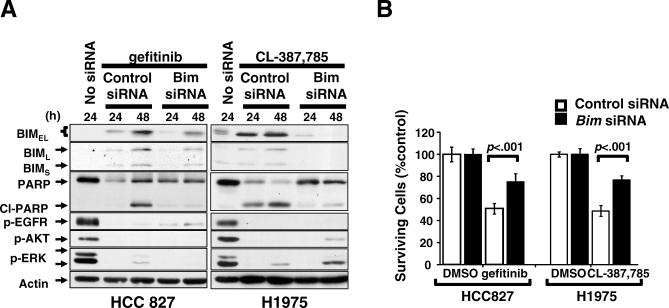
Knockdown of *BIM* Expression Leads to Attenuation of Apoptosis (A) HCC827 cells (left) or H1975 cells (right) were transfected with *BIM* siRNA oligonucleotides or control oligonucleotides for 24 h prior to 0.5 μM gefitinib or 1 μM CL387,785 treatment, respectively. The cells were then treated for 24 and 48 h and lysates were collected and proteins were analyzed by immunoblotting. (B) Annexin V apoptosis assay. The cells were treated as above and analyzed after 48 h. The percentage of surviving cells—both Annexin V^−^ and propidium iodide^−^ cells—was compared with 0.1% DMSO control. The data are reported as mean ± standard deviation (SD) (*n* ≥ 3).

If BIM functions as a mediator of TKI-induced apoptosis, knockdown of its expression should have detectable effects on apoptosis. Therefore, we tested the effects of *BIM* siRNA in HCC827 and H1975 cells treated with gefitinib and CL387,785, respectively. Apoptosis was significantly attenuated by *BIM* siRNA in both cell lines detected by PARP cleavage (Figure 4A) and the Annexin V assay (Figure 4B) after exposure to TKIs that induce dephosphorylation of EGFR, AKT, and ERK (Figure 4A).

These data suggest that BIM plays an important role as a death regulator in TKI-induced apoptosis.

### Identification and Characterization of a Novel Secondary Mutation, L747S, in a Gefitinib-Resistant Tumor

**Figure 5:**
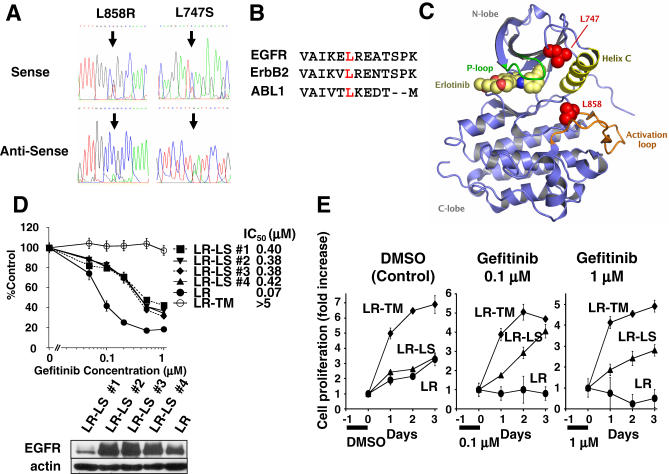
Identification of a Novel Secondary Resistant *EGFR* Mutation, L747S (A) Sequencing chromatograms with the *EGFR* L747S exon 19 and L858R exon 21 by RT-PCR. (B) Amino acid alignments of the tyrosine kinase domain in EGFR, ErbB2, and ABL1. (C) Active conformation crystal structure of the kinase domain of EGFR in complex with erlotinib [34]. The graphic shows the spatial locations of residues L747 and L858 as red spheres. The activation loop is orange, helix αC yellow, the glycine-rich P-loop green, and the inhibitor erlotinib yellow. The figure was made using the program PYMOL (http://pymol.sourceforge.net/). (D) Top: Dose-dependent growth inhibition of Ba/F3 cells expressing EGFR L858R (LR), L858R-T790M (LR-TM), or L858R-L747S (LR-LS) detected by the MTS assay. Error bars indicate standard deviation (*n* = 4). Four LR-LS clones (#1–4) were obtained from G418 selection. Bottom: Expression of EGFR in Ba/F3 cells expressing L858R, L858R-L747S #1–4, or L858R-T790M. (E) Gefitinib release assay. Ba/F3 cells expressing L858R (LR), L858R-L747S #4 (LR-LS), or L858R-T790M (LR-TM) were plated in 24-well plates at a density of 1 × 10^5^/well and treated with 0.1% DMSO (control) or gefitinib for 24 h. Then the cells were washed three times with RPMI and cultured in medium containing 20 ng/ml EGF. The cells were stained with Trypan blue and counted daily. The data are reported as mean ± SD (*n* = 3).

Through the sequencing of gefitinib-resistant tumors at our institution we identified a novel second mutation (see Methods). We detected a L858R *EGFR* mutation in the initial biopsies obtained from a 74-year-old white woman affected by an advanced adenocarcinoma with bronchioalveolar and papillary features [31,32]. She maintained a partial response to gefitinib for 40 months without overt clinical or radiographic progression; however, at that point computer tomography scans showed progression of lung lesions, presence of a thickened left pleura with effusion (Figure S1), and bone metastases. Sequencing of the cDNA derived from the pleural fluid of the progressing tumor confirmed the persistence of the initial L858R mutation in addition to a new T–C basepair change in exon 19 (Figure 5A), which results in a predicted amino acid change of leucine (L) to serine (S) at position 747 of EGFR. Most subclones contained the L747S in cis with L858R (Table S1). In the EGFR kinase domain crystal structures [33,34], L747 is located at the start of the loop between strand β3 and helix αC. This residue is the leucine of the LRE motif that is frequently deleted in exon 19. In crystal structures of both the active and inactive conformations of the EGFR tyrosine kinase domain this residue is oriented toward the back pocket region of the catalytic cleft (Figure 5B). Mutations in the analogous residue of ABL1 (L273M) and ErbB2 (L755S or P) (Figure 5C) have been described in patients with imatinib-resistant chronic myelogenous leukemia (CML) and solid tumors including gastric, breast, and lung cancers, respectively [35–37]. However, functional studies were not performed.

Transient transfection experiments utilizing COS-7 cells demonstrated that auto-phosphorylation of the original L858R EGFR was inhibited by lower concentrations of gefitinib or erlotinib than the L858R-L747S or L858R-T790M constructs (Figure S2A). CL-387,785 partially overcame the observed inhibition (Figure S2B).

To prove the functional significance of the L747S mutation, we generated Ba/F3 cell lines stably expressing mutant EGFR constructs [24]. These cell lines, including L858R-L747S, proliferated in the absence of IL3. However, the proliferation rate of Ba/F3-L858R-L747S cells was not as dramatic as that of Ba/F3-L858R-T790M cells (Figure S3). Ba/F3-L858R cells were extremely sensitive to gefitinib (Figure 5D), whereas Ba/F3-L858R-T790M cells were highly resistant up to 1 μM gefitinib. All four Ba/F3-L858R-L747S clones demonstrated an intermediate pattern of resistance to the growth inhibition signal induced by gefitinib (Figure 5D). After the cells were exposed to gefitinib for 24 h, Ba/F3-L858R cells were unable to proliferate while Ba/F3-L858R-L747S and Ba/F3-L858R-T790M cells continued to grow even in the presence of 1 μM gefitinib (Figure 5E).

These results suggest that execution of apoptosis may be impaired by the presence of T790M and, to a lesser extent, L747S.

### Resistance Mutations Attenuate BIM Up-Regulation and the Mitochondrial Apoptosis Pathway

**Figure 6:**
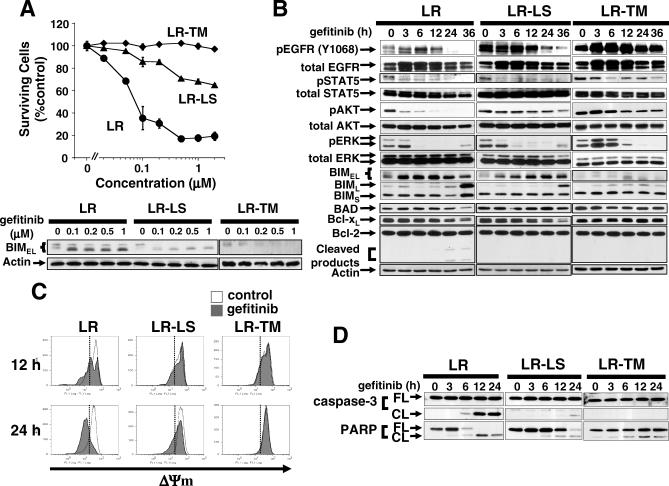
The Secondary Resistant Mutations L747S and T790M Affect Gefitinib-Induced Apoptosis and Inhibit BIM Up-Regulation (A) Top: Annexin V apoptosis assay: Ba/F3 cells expressing L858R (LR), L858R-L747S #4 (LR-LS), or L858R-T790M (LR-TM) cells were grown in the absence or presence of gefitinib for 24 h. The data are reported as mean ± SD (*n* ≥ 3). Bottom: After the cells were treated for 3 h with increasing concentrations of gefitinib, lysates were collected and proteins analyzed by immunoblotting. BIM expression correlated with the amount of apoptosis in L858R, L858R-L747S, and L858R-T790M cells. (B) Modulation of signaling following gefitinib treatment in Ba/F3 cells expressing EGFR mutants. Time course of gefitinib treatment in L858R (LR), L858R-L747S #4 (LR-LS), or L858R-T790M (LR-TM) cells. Cells were treated with 0.2 μM gefitinib for indicated times in the absence of IL3 and in the presence of EGF 20 ng/ml. Note that increase in BIM expression is delayed in LR-LS cells or minimal in LR-TM cells. (C) Flow cytometric analysis of the inner mitochondrial membrane potential (ΔΨ_m_) breakdown. Cells were treated in the presence or absence of 0.2 μM gefitinib for 12 or 24 h, and stained with DiOC6(3). (D) Activation of caspase-3 and cleavage of PARP are attenuated in L858R-L747S #4 (LR-LS) and L858R-T790M (LR-TM) cells. Cell extracts described in (B) were analyzed by Western blotting.

Based on the data obtained from lung cancer cell lines, we further explored the mechanisms by which T790M and L747S resistant mutations affect BIM up-regulation and apoptosis. Gefitinib effectively induced apoptosis in Ba/F3-L858R cells, whereas Ba/F3-L858R-L747S cells were intermediately resistant and Ba/F3-L858R-T790M completely resistant (Figure 6A, top). The up-regulation of BIM was also attenuated in a similar dose–response manner as seen in the apoptosis assay (Figure 6A, bottom), with L858R-T790M completely abrogating the induction of BIM and L858R-L747S attenuating both the up-regulation of BIM and apoptosis.

Phosphorylated EGFR as well as AKT were maintained up to 36 h after gefitinib treatment in Ba/F3-L858R-L747S and L858R-T790M cells. The phosphorylated forms of ERK1/2 were significantly less inhibited in L858R-T790M cells (Figure 6B). Up-regulation of BIM was observed in Ba/F3-L858R cells as early as 3 h after exposure to gefitinib (Figure 6B). Ba/F3-L858R-L747S cells had a delay in the up-regulation of BIM and Ba/F3-L858R-T790M cells had no change in BIM isoforms (Figure 6B). BAD, BCL-x_L_, and BCL2 did not change significantly.

In order to explore the consequences of a delay or inhibition in BIM up-regulation on apoptosis, we examined whether gefitinib induced breakdown of the inner mitochondrial membrane potential (ΔΨ_m_), characterized by a sudden increase in permeability of the mitochondrial membrane as a consequence of the charge difference between the mitochondrial matrix and the cytosol [27,38,39].

Treatment with gefitinib led to a significant decrease in ΔΨ_m_ in Ba/F3-L858R cells at 12 h (Figure 6C). The pan-caspase inhibitor z-VAD-fmk did not inhibit the breakdown of ΔΨ_m_ (unpublished data), suggesting that the intrinsic mitochondrial pathway plays a central role during gefitinib-induced apoptosis. It is well known that the BH3-only members are part of the intrinsic apoptotic pathway [40,41]. Ba/F3-L858R-T790M cells showed no significant change even at 24 h (Figure 6C), and Ba/F3-L858R-L747S cells had no apparent changes at 12 h and only a partial breakdown at 24 h. Activation of caspase-3 and PARP cleavage were consistent with the apoptotic execution process measured by ΔΨ_m_ (Figure 6D).

Taken together, these results suggest that the novel L747S and the T790M mutations attenuate the intrinsic mitochondrial apoptosis pathway by inhibiting the up-regulation of BIM in *EGFR*-mutant models.

## Discussion

We identified BIM as a key apoptotic effector of EGFR TKIs in sensitive cells with the activating L858R or exon 19 deletion EGFR mutations. The common T790M secondary mutation and the novel L747S, in conjunction with an activating mutation, attenuated the up-regulation of BIM and apoptosis.

The discovery that the deregulated tyrosine kinase activity in certain cancers can be targeted has led to major advances in the field of malignant therapeutics [42]. Tyrosine kinases require ATP for their enzymatic activity, and thus small molecules that mimic ATP can bind to mutant or translocated kinases and inactivate them. The most satisfying translational example of this process has been in the case of CML, in which the t(9;22) translocation and the consequent enhanced tyrosine kinase activity of BCR-ABL can be effectively inhibited by imatinib. This oral TKI has revolutionized the treatment of CML and led to sustained responses in a majority of patients [43]. A parallel situation is seen with gastrointestinal stromal tumor (GIST), in which activating mutations of the receptor tyrosine kinases c-KIT or PDGFRA (platelet-derived growth factor receptor, alpha polypeptide) contribute to the disease, and again imatinib is an effective treatment option improving patient survival [44]. A similar clinical benefit is observed in patients with lung cancers harboring the two most common *EGFR* activating mutations, L858R and exon 19 deletions [3–5,9].

The striking initial response to small molecule tyrosine kinase inhibitors is thought to stem from the phenomenon of “oncogene addiction” [21], and a common signaling cascade may be involved in the apoptosis induced by multiple TKIs [22]. Rapid inactivation of phosphorylated ERK, AKT, and STAT3/5, and the delayed accumulation of phosphorylated p38 are commonly observed in SRC-, BCR-ABL-, and EGFR-dependent cells after exposure to the specific inhibitors SU6655, imatinib, and gefitinib, respectively [22].

Recently, it has been shown that BIM is one of the main effectors of imatinib-mediated apoptosis in BCR-ABL-positive leukemia cells [23,45,46]. BIM belongs to the BH3-only group of proteins that bind and neutralize various antiapoptotic BCL2 family members so that they cannot inhibit the proapoptotic effects of BAX and BAK in the intermembrane mitochondrial space [40,41]. BIM is regulated by multiple stimuli, including the PI3K-AKT-FOXO and the ERK1/2 MAPK pathways [29,30]. Cytokine withdrawal or inhibition of PI3K-AKT leads to dephosphorylation and nuclear entry of the forkhead transcription factor FOXO-3A, which induces *BIM* mRNA expression [29]. Inhibition of ERK1/2 also induces *BIM* mRNA expression by unknown mechanisms [29]. In addition, ERK1/2 regulates the function of BIM_EL_ by post-translational modifications. ERK1/2-dependent phosphorylation antagonizes BIM_EL_ by proteasomal degradation or disruption of BIM_EL_-BAX interactions [29].

Of relevance to our studies is that the ErbB family receptors activate the PI3K-AKT and the ERK1/2-MAPK pathways, both of which mediate regulation of BIM. Overexpression of EGFR inhibits anoikis—apoptosis induced by lack of correct cell and extracellular matrix attachment [47]—in mammary epithelial cells by restoring ERK activation and blocking BIM up-regulation, which is reversed by either EGF withdrawal or EGFR inhibition [48]. It has also been shown that EGFR affects the post-translational control of BIM expression through a pathway requiring PKCδ and MEK/MAPK activation [49]. Although the role of p38 as an apoptosis mediator has not been completely characterized [22], p38 activity is required for *EGFR* down-regulation resulting in attenuation of downstream signaling [50]. In addition, p38 activation leads to BIM induction during glucocorticoid-induced apoptosis in lymphoblastic leukemia cells [51].

These observations and the concept of “oncogenic shock,” which postulates that prosurvival signals are shut down quickly, whereas proapoptotic signals remain active enough to induce apoptosis upon oncoprotein inactivation [22], prompted us to hypothesize that BIM plays an important role in apoptosis induced by gefitinib and other EGFR TKIs. Our data indicate that the intrinsic mitochondrial pathway is involved in gefitinib-induced cell death and that the up-regulation of the proapoptotic polypeptide BIM was consistently seen during TKI-induced apoptosis. In addition, sustained activation of EGFR, AKT, and/or ERK caused by the T790M and L747S resistant mutations delayed BIM up-regulation and apoptosis. We also showed, in two distinct cell lines, that knockdown of BIM led to significant reduction in the amount of cell death. These observations suggest that BIM is a key effector of TKI-induced apoptosis in EGFR-driven tumors and that the up-regulation of BIM may be one of the common mechanisms by which tumor cells driven by “oncogenic addiction” undergo apoptosis and “oncogenic shock” [22]. However, knockdown of BIM did not completely inhibit the TKI-induced apoptosis in our cell lines, which may be explained either by the residual level of BIM protein in siRNA transfected cells (Figure 4A), or involvement of other proapoptotic regulators, such as other BH3-only members. One of the possible candidates is BAD, another BH3-only proapoptotic regulator, which was shown previously to play a role in imatinib-induced apoptosis in CML [23]. However, the role of BAD remains unclear so far, since inhibition of BAD phosphorylation, which is believed to be important for preventing apoptosis, was not detected in the NSCLC cells we tested (Figure 3B and unpublished data). In addition, double knockdown of BIM and BAD did not show significant increase in the survival of gefitinib-treated HCC827 cells compared to single BIM knockdown (unpublished data). Further studies are required to define other relevant apoptotic pathways involved in gefitinib-induced apoptosis.

Furthermore, our data indicate that the degree of BIM up-regulation was directly proportional to the amount of apoptosis and that the up-regulation of BIM determines the sensitivity of lung cancer cells to the apoptotic effects of the TKIs. This effect became evident when we examined three cell lines carrying the same EGFR mutation, HCC827, PC-9, and H1650 (all have the delE746-A750 mutation), and noted that the degrees of gefitinib-induced apoptosis were strikingly different, with the former two being more sensitive than the later. The up-regulation of BIM was minimal in H1650 cells, which lack PTEN [52], compared to HCC827 and PC-9.

Acquired resistance to imatinib commonly occurs in CML and often in GIST [53,54]. In both diseases, secondary mutations in either *ABL1* or *KIT* and *PDGFRA* have been identified as main mechanistic factors that re-establish the oncogene signaling in these tumors. In the case of CML, more than 35 mutations have been described in the ABL kinase domain from patients with imatinib resistance [55], and in GIST a similar pattern of multiple sites of secondary kinase domain mutations has been seen [54]. There are many similarities among structures of tyrosine kinases, and some of the secondary mutations fall at exactly the same amino acid residue. This is the case of the T315I, T670I, and T790M mutations in *ABL1*, *KIT*, and *EGFR*, respectively [56]. Mutation of these key gatekeeper residues can prevent inhibitor access to the kinase back pocket region and can disrupt hydrogen bonds or other interactions between the inhibitor and the kinase [16,56]. Despite the similarity between CML, GIST, and *EGFR*-mutated NSCLC in the patterns of secondary resistance, in NSCLC only the T790M [16,17] and D761Y [19] secondary mutations have been described so far.

In this report, we identify and characterize a novel EGFR secondary mutation, L747S. Our in vitro studies demonstrated that both of the secondary mutations initially identified in our lab (T790M and L747S) confer varying degrees of resistance to the apoptotic signals initiated by gefitinib. These changes may reflect the progressing radiological and clinical pictures of our patients while on gefitinib monotherapy for their metastatic NSCLC. T790M results in ineffective TKI inhibition and sustained down-stream signaling from the mutant *EGFR* [16,17], as was seen in all cell lines carrying T790M in the current report. EGFR, AKT, and ERK1/2 were not inhibited in our T790M gefitinib-treated models. The L747 residue is oriented toward the back pocket region of the catalytic cleft both in the active and inactive conformation of the EGFR tyrosine kinase domain [33,34]. It is unclear from the current crystallographic data and from our molecular dynamics simulations how acquisition of this mutation results in resistance to ATP-competitive EGFR inhibitors; there may be differential effects on ATP and small molecule binding, as seen for L858R and G719S mutations [57] or a shift in the conformational equilibrium of the kinase between the active and inactive states. Further crystallographic studies are required to more fully understand the structural basis for the effects of this mutation.

Our data show that L858R-L747S demonstrated a pattern of resistance that was less pronounced than that observed with L858R-T790M with increasing doses of gefitinib (Figures 5D and 6A). These results are similar to the ones observed in the previously reported secondary L858R-D761Y mutation [19]. It is possible to conceive that an increase in the clinical doses of gefitinib or switching to erlotinib, which is given at its maximal tolerated dose [58], may lead to beneficial clinical effects, possibly by increasing BIM expression, in patients with *EGFR* mutations who acquired L747S after exposure to gefitinib.

The data presented here indicate that BIM is both a marker and an effector of TKI-induced apoptosis in *EGFR*-mutant NSCLC cells. Furthermore, we identified a novel acquired *EGFR* secondary mutation, L747S, and showed that both the L747S and the common T790M in cis to an activating *EGFR* mutation (either L858R or an exon 19 deletion) cause resistance to EGFR TKI-induced apoptosis and attenuate the up-regulation of BIM. In this context, it is possible that enhancement of BIM expression or activation of its downstream targets may be a promising strategy for the treatment of *EGFR*-mutant NSCLC, particularly in the context of mutations conferring secondary resistance to TKI inhibitors.

## Supporting Information

Figure S1 Chest CT Scans Before (Left) and After (Right) Progression of Lung Tumors Note that pleural effusion was detected in left cavity.
(330 KB PPT)

Figure S2 The EGFR-L747S-L858R Double Mutant Is Less Sensitive to Inhibition by Gefitinib and Erlotinib (A) Autophosphorylation of EGFR tyrosine 1068 is detected by immunoblots of whole-cell extracts isolated from transfected COS-7 cells after a 3-h incubation with different concentrations of gefitinib. Total EGFR expression is shown as loading control.
(B) The inhibition of EGFR autophosphorylation by CL-387,785. Blots were probed with EGFR tyrosine 1068 (left) and total EGFR antibody (right).
(398 KB PPT)

Figure S3 Functional Analyses of Ba/F3 Cells Expressing *EGFR* Mutants Top: Expression of EGFR in Ba/F3 cells expressing L858R (LR), L858R-L747S (LR-LS#4), wild-type EGFR (WT), L747S (LS), or L858R-T790M (LR-TM). Bottom: IL3-independent growth of Ba/F3 cells expressing the EGFR mutants. Cells were seeded at a density of 1 × 10^4^/ml and counted daily.
(4.4 MB PPT)

Table S1 Characteristics and Clinical Course of Patients with Gefitinib-Resistant *EGFR*-Mutant NSCLCs and Secondary *EGFR* Mutations from the Thoracic Oncology Clinic at Beth Israel Deaconess Medical Center (28 KB DOC)

### Accession Numbers

The NCBI GenBank (http://www.ncbi.nlm.nih.gov/) accession numbers for the genes discussed in this paper are *BAD* (NM_032989), *BIM* (AF032458), and *EGFR* (NM_005228). The Protein Data Bank (http://www.pdb.org/) accession number for the EGFR tyrosine kinase domain with the 4-anilinoquinazoline inhibitor erlotinib is 1M17.

## Abbreviations

CML: chronic myelogenous leukemia
EGFR: epidermal growth factor receptor
ERK: extracellular signal-regulated protein kinase
GIST: gastrointestinal stromal tumors
MAPK: mitogen-activated protein kinase
NSCLC: non-small cell lung cancer
PARP: poly (ADP-ribose) polymerase
SD: standard deviation
siRNA: small interfering RNA
TKI: tyrosine kinase inhibitor
WT: wild type
