# Supplementary material for: BIM Mediates EGFR Tyrosine Kinase Inhibitor-Induced Apoptosis in Lung Cancers with Oncogenic EGFR Mutations
Source: PLoS Med. 2007 Oct 30;4(10):e315. doi: 10.1371/journal.pmed.0040315 (PMC2043012; doi:10.1371/journal.pmed.0040315)
Supplement: Figure S1 — Note that pleural effusion was detected in left cavity. (330 KB PPT) [file pmed.0040315.sg001.ppt]

## Slide 1
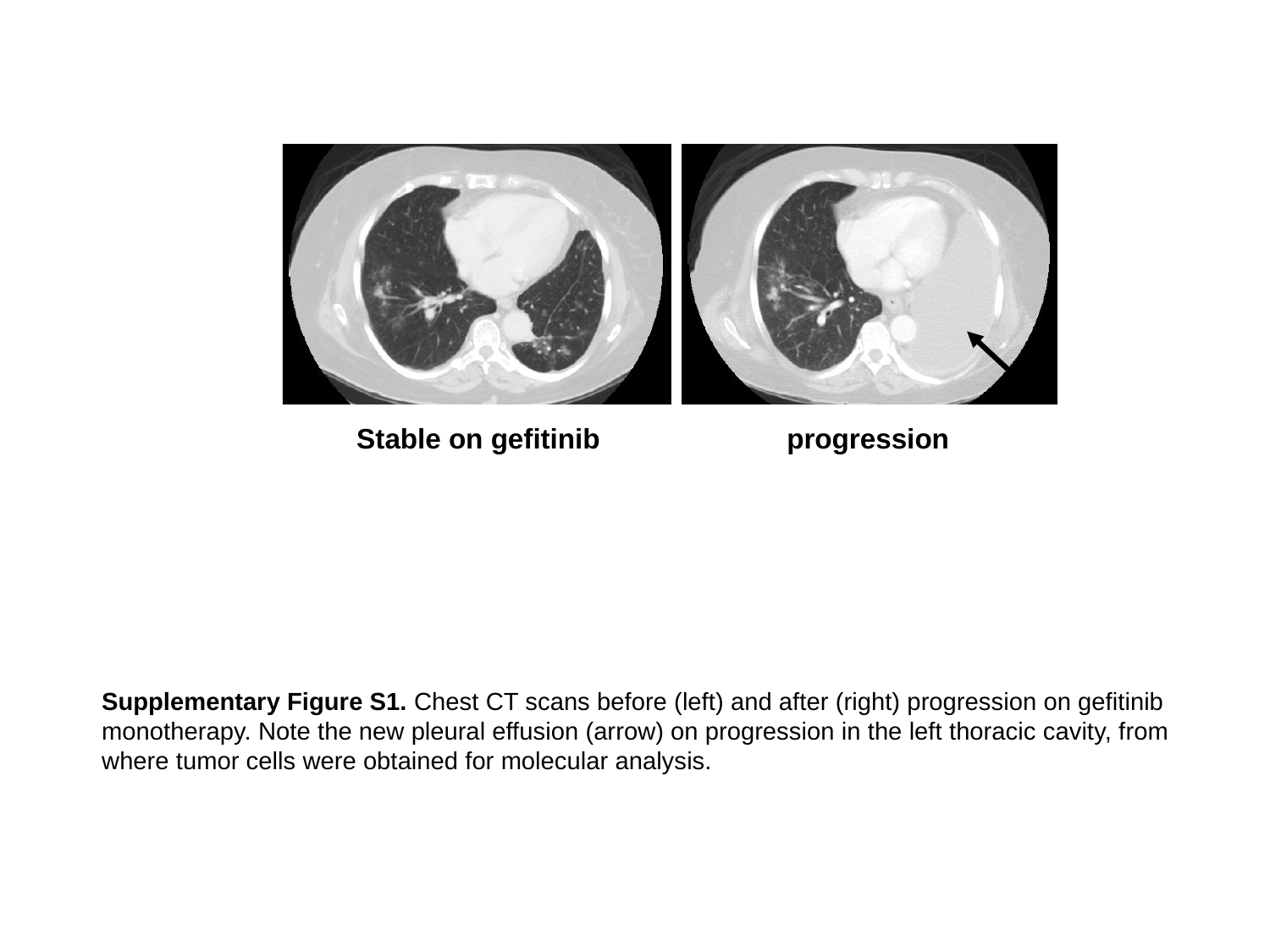

Stable on gefitinib
progression
Supplementary Figure S1. Chest CT scans before (left) and after (right) progression on gefitinib monotherapy. Note the new pleural effusion (arrow) on progression in the left thoracic cavity, from where tumor cells were obtained for molecular analysis.
