# Supplementary material for: BIM Mediates EGFR Tyrosine Kinase Inhibitor-Induced Apoptosis in Lung Cancers with Oncogenic EGFR Mutations
Source: PLoS Med. 2007 Oct 30;4(10):e315. doi: 10.1371/journal.pmed.0040315 (PMC2043012; doi:10.1371/journal.pmed.0040315)
Supplement: Figure S2 — (A) Autophosphorylation of EGFR tyrosine 1068 is detected by immunoblots of whole-cell extracts isolated from transfected COS-7 cells after a 3-h incubation with different concentrations of gefitinib. Total EGFR expression is shown as loading control. (B) The inhibition of EGFR autophosphorylation by CL-387,785. Blots were probed with EGFR tyrosine 1068 (left) and total EGFR antibody (right). (398 KB PPT) [file pmed.0040315.sg002.ppt]

## Slide 1
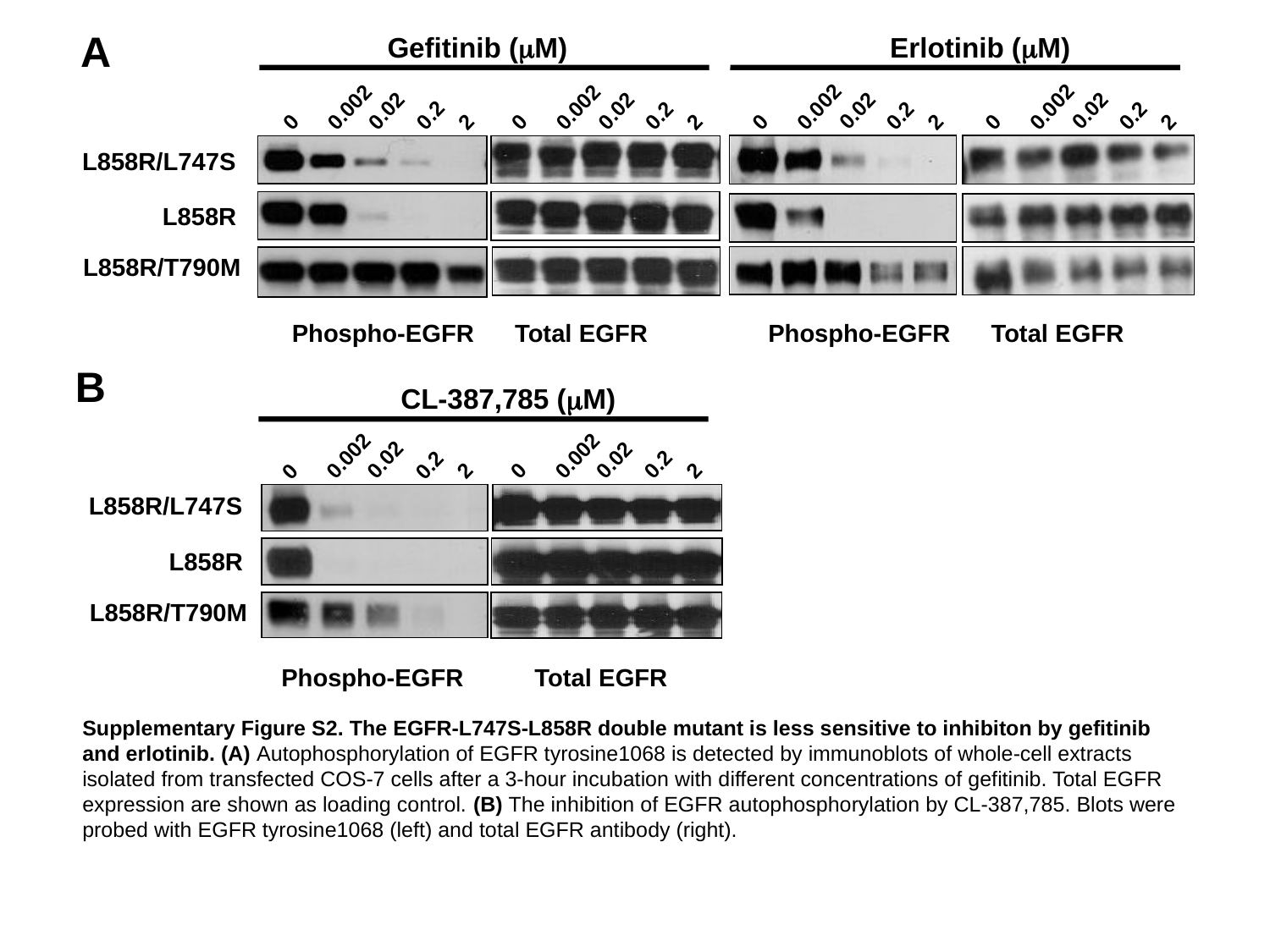

A
Gefitinib (μM)
Erlotinib (μM)
0.002
0.002
0.002
0.002
0.02
0.02
0.02
0.02
0
0
0
0
0.2
2
2
2
0.2
2
0.2
0.2
L858R/L747S
L858R
L858R/T790M
Phospho-EGFR
Total EGFR
Phospho-EGFR
Total EGFR
B
CL-387,785 (μM)
0.002
0.002
0.02
0.02
0
0
0.2
2
2
0.2
L858R/L747S
L858R
L858R/T790M
Phospho-EGFR
Total EGFR
Supplementary Figure S2. The EGFR-L747S-L858R double mutant is less sensitive to inhibiton by gefitinib and erlotinib. (A) Autophosphorylation of EGFR tyrosine1068 is detected by immunoblots of whole-cell extracts isolated from transfected COS-7 cells after a 3-hour incubation with different concentrations of gefitinib. Total EGFR expression are shown as loading control. (B) The inhibition of EGFR autophosphorylation by CL-387,785. Blots were probed with EGFR tyrosine1068 (left) and total EGFR antibody (right).
