# Supplementary material for: BIM Mediates EGFR Tyrosine Kinase Inhibitor-Induced Apoptosis in Lung Cancers with Oncogenic EGFR Mutations
Source: PLoS Med. 2007 Oct 30;4(10):e315. doi: 10.1371/journal.pmed.0040315 (PMC2043012; doi:10.1371/journal.pmed.0040315)
Supplement: Figure S3 — Top: Expression of EGFR in Ba/F3 cells expressing L858R (LR), L858R-L747S (LR-LS#4), wild-type EGFR (WT), L747S (LS), or L858R-T790M (LR-TM). Bottom: IL3-independent growth of Ba/F3 cells expressing the EGFR mutants. Cells were seeded at a density of 1 × 10^4^/ml and counted daily. (4.4 MB PPT) [file pmed.0040315.sg003.ppt]

## Slide 1
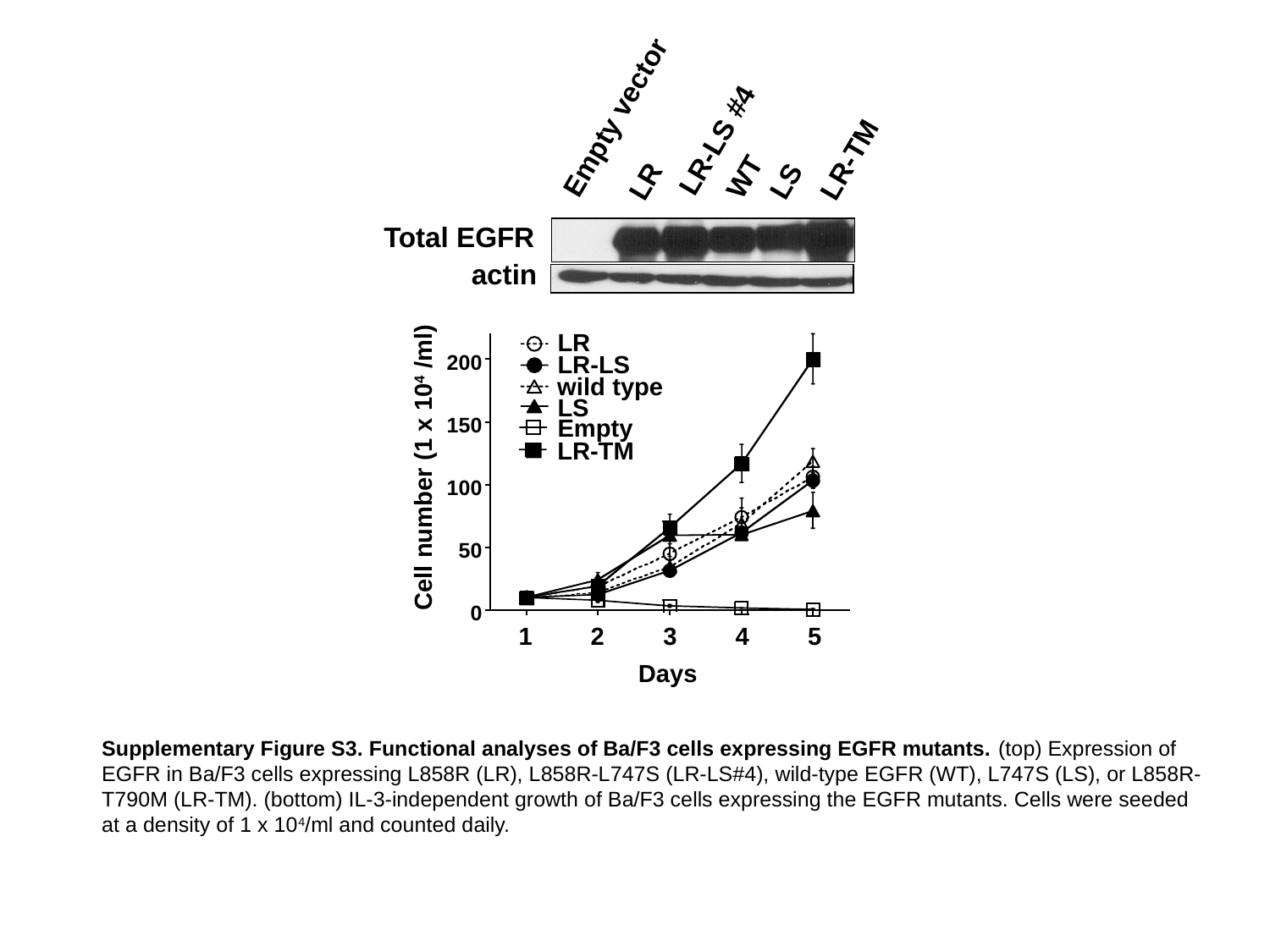

Empty vector
LR-LS #4
WT
LS
LR-TM
LR
Total EGFR
actin
LR
LR-LS
200
wild type
LS
150
Empty
LR-TM
Cell number (1 x 10^4^ /ml)
100
50
0
1
2
3
4
5
Days
Supplementary Figure S3. Functional analyses of Ba/F3 cells expressing EGFR mutants. (top) Expression of EGFR in Ba/F3 cells expressing L858R (LR), L858R-L747S (LR-LS#4), wild-type EGFR (WT), L747S (LS), or L858R-T790M (LR-TM). (bottom) IL-3-independent growth of Ba/F3 cells expressing the EGFR mutants. Cells were seeded at a density of 1 x 10^4^/ml and counted daily.
