# Supplementary material for: BIM Mediates EGFR Tyrosine Kinase Inhibitor-Induced Apoptosis in Lung Cancers with Oncogenic EGFR Mutations
Source: PLoS Med. 2007 Oct 30;4(10):e315. doi: 10.1371/journal.pmed.0040315 (PMC2043012; doi:10.1371/journal.pmed.0040315)
Supplement: Table S1 — (28 KB DOC) [file pmed.0040315.st001.doc]

**SUPPLEMENTARY TABLE**

**Characteristics and clinical course of patients with gefitinib-resistant EGFR-mutant NSCLCs and secondary EGFR mutations from the Thoracic Oncology Clinic at Beth Israel Deaconess Medical Center**

| **Patient** | **Age**  **(yrs)** | **Sex** | **Histology/Stage** | **Smoking History** | **Therapy prior to gefitinib** | **EGFR sequence of original specimen** | **Response to gefitinib (250 mg/day)** | **TTP**  **(in months)** | **EGFR sequence^*^ of gefitinib-resistant tumor (source)** | **Subsequent therapies** | **Overall survival (in months, from gefitinib)** |
| --- | --- | --- | --- | --- | --- | --- | --- | --- | --- | --- | --- |
| 1 | 71 | M | adenocarcinoma with bronchioalveolar features/  IIIB → IV | former  (40 pack-years)^+^ | carboplatin, paclitaxel, docetaxel, gemcitabine | L747-S752 deletion | CR | 24 | L747-S752 deletion + T790M  (transbronchial biopsy) ^§^ | cetuximab, erlotinib, experimental Raf kinase inhibitor | 30 |
| 2 | 74 | F | adenocarcinoma with bronchioalveolar and papillary features/  IIB →IV | non-smoker | surgery, XRT, carboplatin, paclitaxel | L858R | PR | 40 | L858R + L747S  (pleural effusion) ^¶^ | experimental EGFR/ErbB2 inhibitor, erlotinib (150mg/day) | > 50 |

EGFR, epidermal growth factor receptor; NSCLC, non-small cell lung cancer; yrs, years; ref, reference; M, male; F, female; yrs, years; XRT, radiation therapy; CR, complete response; PR, partial response; TTP, time to progression; ^*^ exon 18 to 21 of the EGFR were sequenced from DNA and RNA (cDNA); ^+^ patient smoked 1 pack per day for 40 years and had quit > 10 years prior to diagnosis; ^§^ subcloning of cDNA: 5/40 L747-S752del-T790M, 18/40 WT/WT, 9/40 WT-T790M, 8/40 L747-S752del-WT ; ^¶^ subcloning of cDNA: 12/17 L858R-L747S, 3/17 WT-WT, 1/17 L858R-WT, 1/17 WT-L747S.
